# Data-Driven and Machine-Learning Methods to Project Coronavirus Disease 2019 Pandemic Trend in Eastern Mediterranean

**DOI:** 10.3389/fpubh.2021.602353

**Published:** 2021-05-13

**Authors:** Wenbo Huang, Shuang Ao, Dan Han, Yuming Liu, Shuang Liu, Yaojiang Huang

**Affiliations:** ^1^Beijing Engineering Research Center of Food Environment and Public Health, Minzu University of China, Beijing, China; ^2^Department of Software and Information, Beijing Information Technology College, Beijing, China; ^3^College of Medicine, Minzu University of China, Beijing, China; ^4^College of Life and Environmental Sciences, Minzu University of China, Beijing, China; ^5^Harvard T.H. Chan School of Public Health, Boston, MA, United States

**Keywords:** COVID-19, data-driven, machine learning, assessment, projection

## Abstract

**Background:** The coronavirus disease 2019 (COVID-19) pandemic has become a major public health crisis worldwide, and the Eastern Mediterranean is one of the most affected areas.

**Materials and Methods:** We use a data-driven approach to assess the characteristics, situation, prevalence, and current intervention actions of the COVID-19 pandemic. We establish a spatial model of the spread of the COVID-19 pandemic to project the trend and time distribution of the total confirmed cases and growth rate of daily confirmed cases based on the current intervention actions.

**Results:** The results show that the number of daily confirmed cases, number of active cases, or growth rate of daily confirmed cases of COVID-19 are exhibiting a significant downward trend in Qatar, Egypt, Pakistan, and Saudi Arabia under the current interventions, although the total number of confirmed cases and deaths is still increasing. However, it is predicted that the number of total confirmed cases and active cases in Iran and Iraq may continue to increase.

**Conclusion:** The COVID-19 pandemic in Qatar, Egypt, Pakistan, and Saudi Arabia will be largely contained if interventions are maintained or tightened. The future is not optimistic, and the intervention response must be further strengthened in Iran and Iraq. The aim of this study is to contribute to the prevention and control of the COVID-19 pandemic.

## Introduction

Half a year has passed since the WHO announced the coronavirus disease 2019 (COVID-19) pandemic, which has not disappeared because of climate and other factors. Instead, the epidemic has spread to every corner of the world and is worsening. Obviously, the COVID-19 pandemic has no chance of ending before the end of 2020 (1–3). Countries around the world have successively implemented pharmaceutical and non-pharmaceutical intervention measures to control the COVID-19 pandemic, but there are no special and specific drugs available for treatment. The intervention measures focus on isolation of suspected and confirmed cases, movement restrictions and social distancing, contact tracing, public health measures, and lockdowns (4–6), but the COVID-19 pandemic continues to spread in most countries.

In recent years, a large amount of theoretical and applied research evidence has shown that mathematical projection and modeling play a key role in understanding disease dynamics and transmission and finding the best intervention strategies for infectious diseases (7–15). The COVID-19 pandemic has become a major public health crisis worldwide, and it has severely affected the economies, employment, and livelihoods of all countries. The Eastern Mediterranean is one of the regions most affected by COVID-19 (2, 3, 16, 17). In the Eastern Mediterranean, oil exports have dropped significantly and income has fallen sharply this year, public health resources are relatively scarce, the population is large, and the economy is relatively underdeveloped (18). At the same time, in some countries, COVID-19 epidemiological data are inaccurate; or difficulty in identifying cases, underreporting, and misdiagnosis are problems. This poses a huge challenge to the prevention and control of COVID-19 in these countries (9, 12, 19–25). Therefore, it is especially important to use data-driven modeling to evaluate the current situation and the effectiveness of intervention measures in the uncertain stage of the COVID-19 epidemic and to use artificial intelligence to predict the trend of the pandemic in Eastern Mediterranean countries.

Data-driven methods were used in this study for evaluation, and machine-learning methods were used to predict the COVID-19 pandemic for the six countries with the largest number of COVID-19 confirmed cases in the Eastern Mediterranean. The purpose was to evaluate the *status quo* to conduct a model of COVID-19 spread, as well as to project the trend and time distribution of the total confirmed cases and the single-day confirmed cases of COVID-19 in those countries. The purpose of this work is to promote further applications and thereby help prevent and control the COVID-19 pandemic.

## Materials and Methods

This study was conducted through data download, status assessment, the non-pharmaceutical intervention actions and epidemic trend prediction, etc. The flowchart of the method is shown in Supplementary Figure 1.

### Data Sources

Data from August 20, 2020, in six countries with the highest number of confirmed COVID-19 cases in the Eastern Mediterranean were studied. The data were downloaded from Johns Hopkins CSSE (https://github.com/CSSEGISandData/COVID-19/tree/master/csse_covid_19_data), COVID-19 Government Measures Dataset (5), and Oxford COVID-19 Government Response Tracker (OxCGRT).

### Evaluation of the Coronavirus Disease 2019 Pandemic in Eastern Mediterranean

The current status of the COVID-19 epidemic includes the total number of confirmed cases, deaths, active cases, overall growth rate, total number of confirmed cases per million people, and number of daily changes in each country. These were evaluated using the R (26) package COVID19. Analytics and EpiModel based on the current intervention actions.

### Non-pharmaceutical Intervention Actions

The R was used to analyze the OxCGRT intervention action data (Supplementary Material 1), which provide a composite of nine measures: school closures, stay-at-home requirements, workplace closures, restrictions on public gatherings, public information campaigns, closures of public transport, restrictions on internal movements, cancellation of public events, and international travel controls. The score of the nine measures was between 0 and 100 on any given day. This index indicates the strict government and societal response.

### Forecasting the Trend of the Coronavirus Disease 2019 Pandemic in Eastern Mediterranean

The trend of the total confirmed COVID-19 cases was forecast based on existing intervention actions, and a 180-day-ahead forecast was performed with a 95% prediction interval (PI) using machine learning with Python Prophet Module (27) in Iran, Saudi Arabia, Pakistan, Iraq, Qatar, and Egypt. The machine-learning forecast model was utilized with additional regression elements and no tweaking of season-related parameters. Machine-learning methods with Python Prophet module (27, 28) were used to project the daily growth rate in each country and generate a 180-day-ahead forecast with a 95% PI. The calculation formula and algorithm is as follows:

$$\begin{array}{l} y\left(t\right)=g\left(t\right)+s\left(t\right)+h\left(t\right)+\epsilon_{t} \end{array}$$

A basic model was established with additional regression elements. Machine learning with the Python Prophet module assigns a predicted value for each day in the future, named Yhat_lower, Yhat, and Yhat_upper, which are, respectively, the predicted Yhat and the lower and upper Yhat of the projection with a 95% PI.

## Results

### Evaluation of the Coronavirus Disease 2019 Pandemic in Eastern Mediterranean

#### Status Quo of the Coronavirus Disease 2019 Pandemic

**Figure 1:**
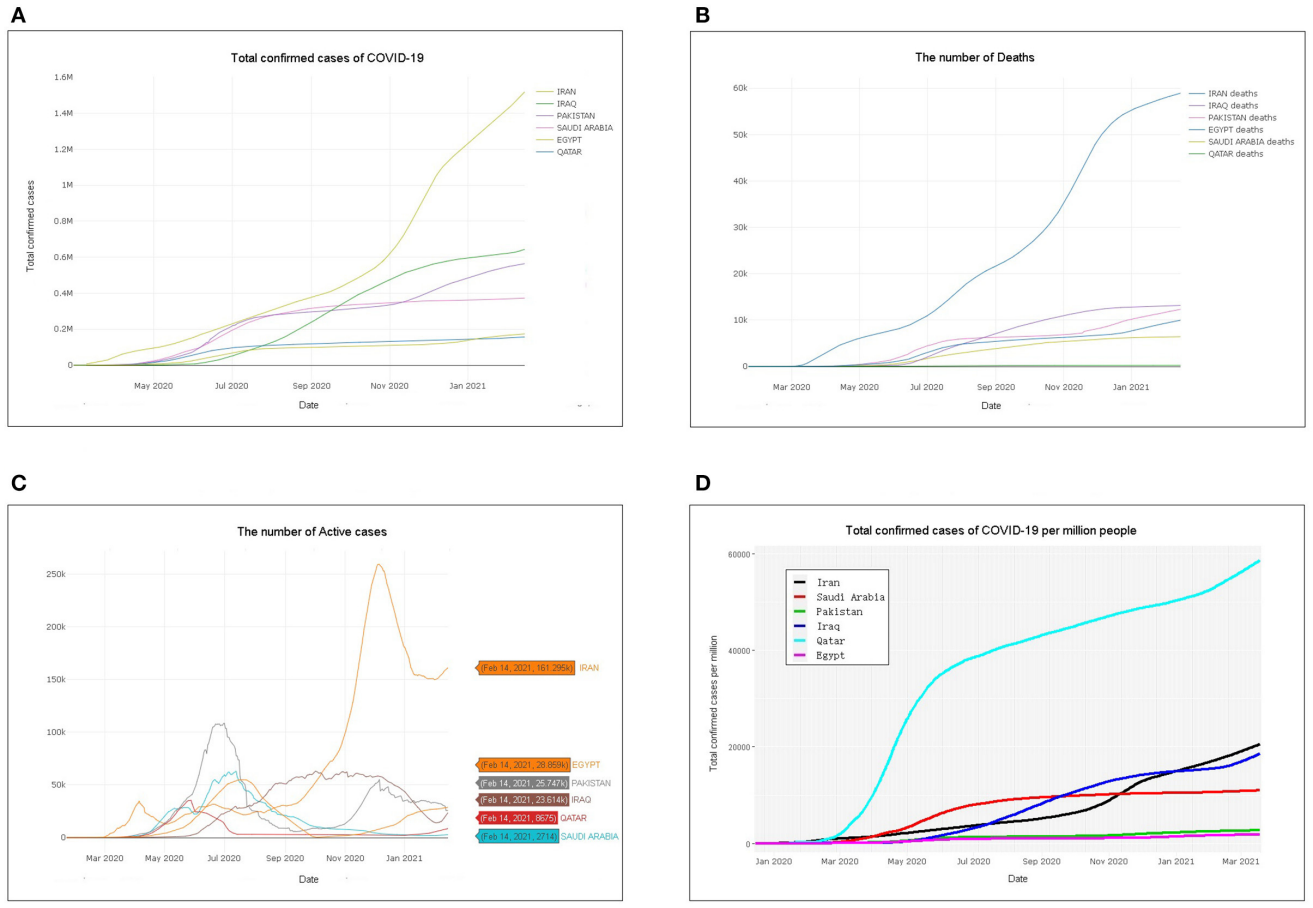
Total confirmed cases, deaths, active cases, and total confirmed cases per million people: **(A**) number of total confirmed coronavirus disease 2019 (COVID-19) cases, **(B)** number of deaths in six Eastern Mediterranean countries, **(C)** number of active cases in six Eastern Mediterranean countries, and **(D)** total confirmed cases per million people.

As of February 14, 2021, the six countries with the highest number of COVID-19 cases in the Eastern Mediterranean were Iran, Saudi Arabia, Pakistan, Iraq, Qatar, and Egypt. Iran had 1.518263 *M* confirmed cases, 1.298032 *M* recovered, 58.945 *k* deaths, and 161.295 *k* active cases with the rising curve of the wave (Figures 1A–C and Supplementary Figure 2a). The numbers of confirmed cases, recovered patients, deaths, and active cases were 564.077, 525.997, 12.333, and 25.747 *k* in Pakistan, with the number of active cases showing a wave decreasing trend. In Saudi Arabia, 372.732 *k* confirmed cases, 363.585 *k* recovered, 2,714 deaths, and 6,433 active cases with downward trends were reported. There were 643.852 *k* confirmed cases, 607.059 *k* recovered, 13.179 *k* deaths, and 23.614 *k* active cases that developed in waves in Iraq (Figure 1 and Supplementary Figure 2d). Egypt had 173.813 *k* confirmed cases, 134.96 *k* recovered, 9.994 *k* deaths, and 28.859 *k* active cases that developed in waves and rose after November 2020. In Qatar, 157.244 *k* confirmed cases, 148.314 *k* recovered, 255 deaths, and 8,675 active cases with decreasing trend were confirmed (Figures 1A–C and Supplementary Figure 2).

The total number of confirmed cases per million people in the ongoing COVID-19 pandemic was estimated. Qatar had the highest number of confirmed cases per million people, followed by Saudi Arabia, Iraq, Pakistan, Iraq, Qatar, and Egypt, which was the lowest (Figure 1D). The overall growth rates and the total number of cases were evaluated with a confidence band based on the moving average for different countries. The number of cases as a function of time was used to generate different fits to match the data in a linear-scale and log-scale plot for the given locations and types. If the overall growth rate is close to 1, it indicates that the spread of the virus has reached its logical asymptote. In other cases, as in the six countries in the Eastern Mediterranean on the date used in this study, it is still higher than 1, indicating that the total number of confirmed cases continues to grow (lm-exp GR: 1.02–1.03, glm-Poisson GR: 1.01, and Supplementary Figures 3a–f).

#### Daily Changes in the Coronavirus Disease 2019 Pandemic

**Figure 2:**
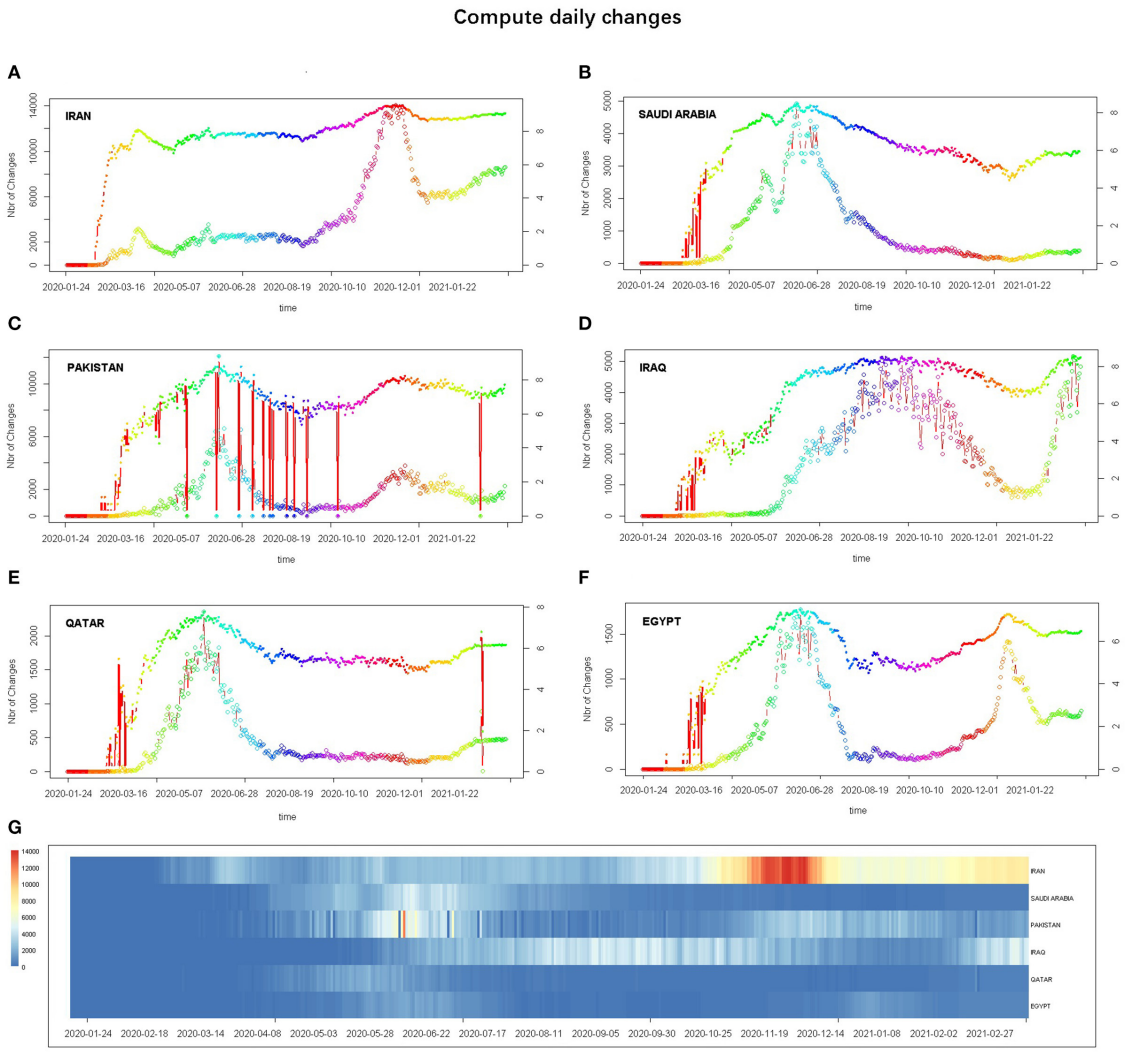
Evaluated daily changes in the number of confirmed cases: **(A–F)** subgraph showing the daily changes Iran, Saudi Arabia, Pakistan, Iraq, Qatar, and Egypt, respectively—the daily changes in the number of confirmed cases in linear scale (bottom thin line, left *y*-axis) and log scale (upper thicker line, right *y*-axis)—and **(G)** heatmap drawn to compare daily changes in confirmed cases (horizontal axis) in the different countries (vertical axis).

Daily changes in confirmed cases were evaluated by plotting two scatter plots in log scale (right vertical axis) and linear scale (left vertical axis) with the number of changes, and a mosaic-type layout heatmap comparing daily changes in confirmed cases in six countries was plotted (Figure 2G). As of February 14, 2021, the daily number of confirmed cases in Iran showed multiple peaks with an overall upward trend, whereas the daily change in Iraq continued to rise (Figures 2A,D,G). Saudi Arabia, Pakistan, Qatar, and Egypt had multiple peaks in the number of daily confirmed cases and showed a downward trend (Figures 2B,C,E–G).

### Non-pharmaceutical Intervention Actions

**Figure 3:**
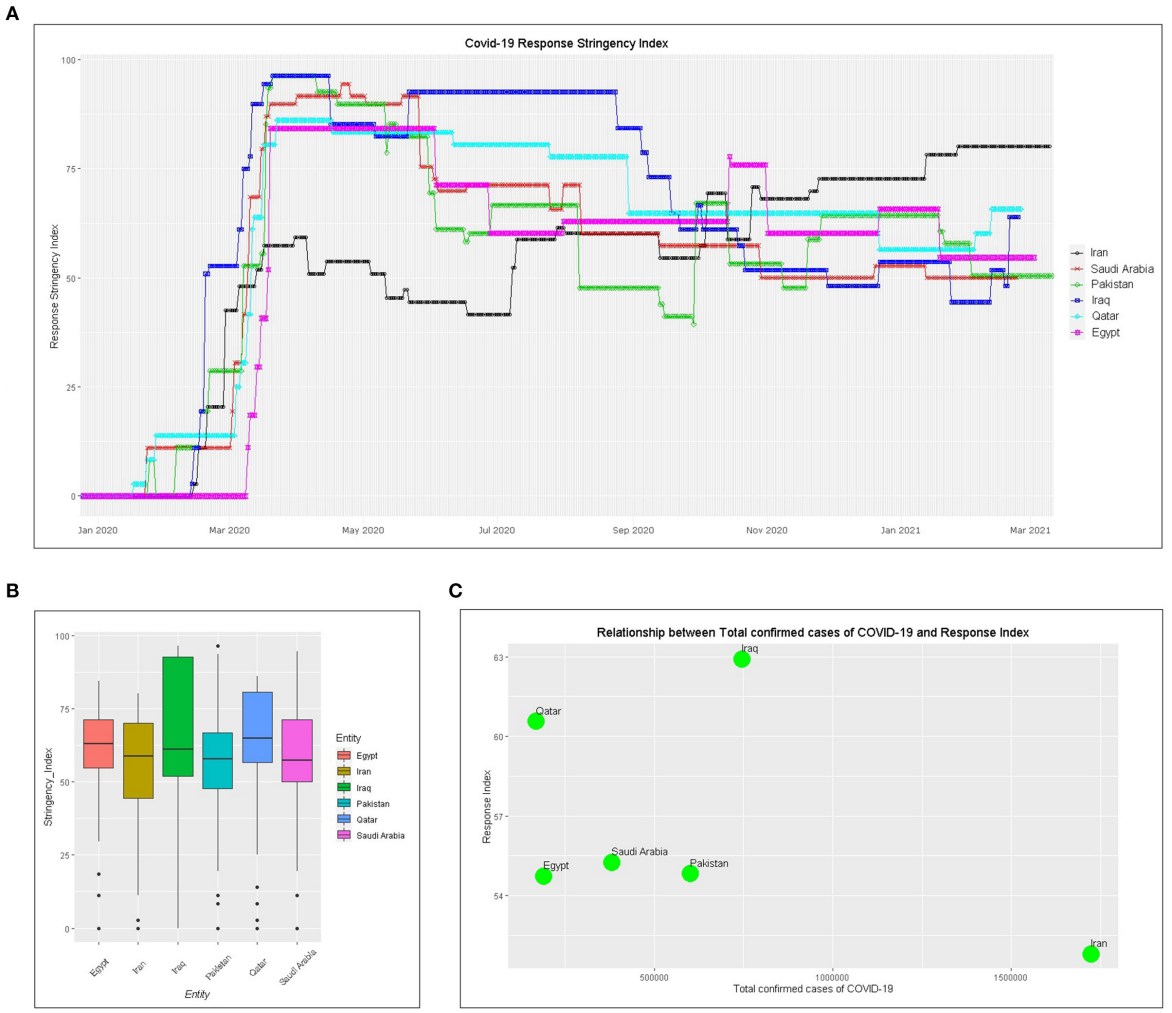
Government intervention index and its relationship to confirmed cases: **(A)** government or societal intervention index, **(B)** bar plot of intervention score since January 2020, and **(C)** relationship between government response index and the number of confirmed cases.

The OxCGRT government intervention index data were analyzed to evaluate the level of government and social response using the R tidycovid19 package. The higher the score of the index, the stricter the government and societal response. Six countries began implementing strict intervention measures at approximately the same time. Since late March, Iran's intervention index has been approximately 50, while other countries have relatively high levels of intervention (Figures 3A,B). The mean of intervention measures score since January shows that Iraq has the highest, followed by Qatar, Saudi Arabia, Pakistan, and Egypt, and Iran has the lowest (Figures 3B,C). Although Iran's regulatory index rose to first place around November 2020, the total number of confirmed cases was the highest (Figure 3C).

### Forecasting the Trend of the Coronavirus Disease 2019 Pandemic in Eastern Mediterranean

**Figure 4:**
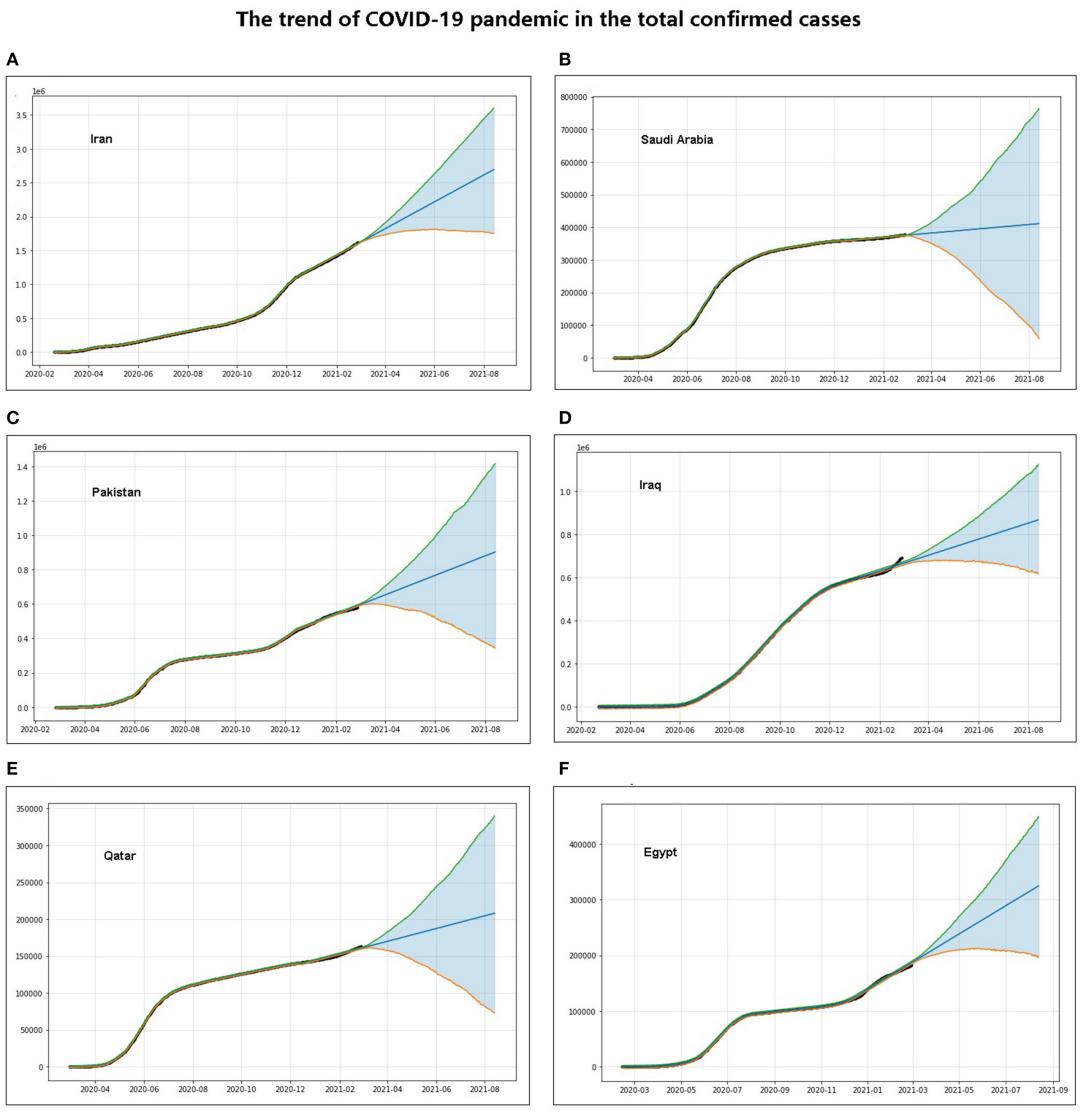
Forecast of overall trends in the coronavirus disease 2019 (COVID-19) pandemic: **(A–F)** trends of the number of total COVID-19 confirmed cases forecast by machine-learning methods in Iran, Saudi Arabia, Pakistan, Iraq, Qatar, and Egypt, respectively.

The total confirmed cases and daily growth rate of the COVID-19 pandemic were projected using machine learning with additional regression elements and no tweaking of season-related parameters. A forecast value was assigned for each day in the future, namely, Yhat_upper, Yhat, and Yhat_lower. In terms of the growth trend of the total number of confirmed cases of COVID-19, as of August 13, 2021, Iran's Yhat, Yhat_lower, and Yhat_upper values are predicted to be 2,693.448, 1,799.289, and 3,630.919 *k*, respectively. The predicted Yhat value for Egypt is 324.325 *k*, and Yhat_upper and Yhat_lower are 448.601 and 196.262 *k*, respectively, in the total number of confirmed cases of COVID-19. Iraq's Yhat, Yhat_lower, and Yhat_upper values are predicted to be 867.794, 616.828, and 1,125.184 *k*, respectively (Figure 4 and Supplementary Table 1). Under existing intervention actions (Figure 3), machine-learning projections show that Saudi Arabia (Yhat: 410.981 *k*, PI: 59.156–763.790 *k*), Pakistan (Yhat: 901.943 *k*, PI: 344.972–1,415.126 *k*), and Qatar (Yhat: 207.910 *k*, PI: 73.118–339.932 *k*) will be flat in the total number of confirmed cases of COVID-19, while it does not seem to be slowing down in Iran and Egypt (Figure 4 and Supplementary Table 1).

**Figure 5:**
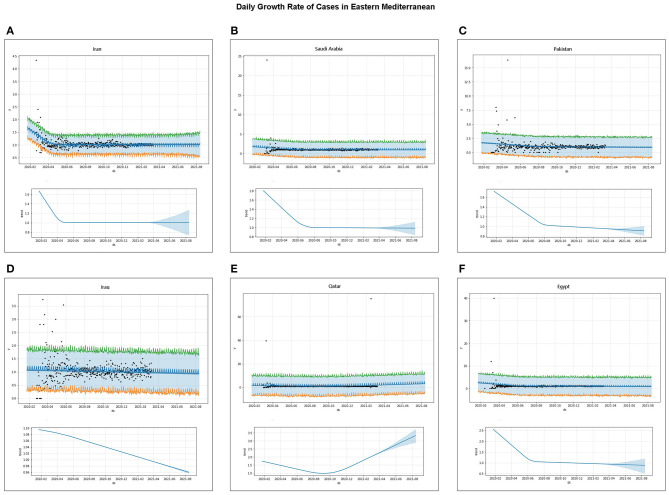
Forecast of trends in daily confirmed cases of the coronavirus disease 2019 (COVID-19) pandemic: **(A–F)** trends of the daily number of confirmed cases forecast by machine learning in Iran, Saudi Arabia, Pakistan, Iraq, Qatar, and Egypt, respectively—the top panel forecasts the daily number of confirmed cases obtained by machine learning, the bottom panel forecasts the trend of the number of daily confirmed cases, the *ds*-coordinates in the figure represent the date, and the *y*-coordinates correspond to the predicted values.

Under the circumstance that the severity of the existing interventions remains unchanged, Saudi Arabia, Pakistan, Iraq, and Egypt all showed a downward trend in the growth rate of daily confirmed cases, whereas Iran and Qatar showed no significant downward trend in the growth rate of daily confirmed cases (Figures 5A–E). The growth rate of the daily confirmed cases in Iraq is going to be <1 around March 2021, that in Pakistan is going to be <1 around January 2021, and that in Saudi Arabia and Egypt is going to be <1 around February 2021 (Figures 5B–D,F).

## Discussion

The current status of the COVID-19 pandemic was evaluated. Iran has the highest total number of COVID-19 confirmed cases, while Qatar has the highest number of confirmed cases of COVID-19 per million people (Figures 1A,D). The number of active cases in Pakistan peaked at 108,642 on July 1. In Qatar, it is also declining with a small active case stock, which indicates that the epidemic is largely under control in both countries. The number of active cases in both Saudi Arabia is on a downward trend, suggesting that the epidemic should improve. The active cases in Iran, Iraq, and Egypt show a small rise in waves with a large stock, which is not optimistic (Figure 1C). Saudi Arabia, Pakistan, and Qatar have all peaked and show a downward trend in the daily number of confirmed cases, while Iran, Iraq, and Egypt show a wave (Figures 2A,D,F). It is worth noting that the number of active cases and daily changes in all countries showed a slight upward trend toward the end of 2020 or the beginning of 2021 (Figures 1C, 2), which may be related to the reduction of non-pharmaceutical intervention actions in these countries starting in September 2020 (Figure 3). Although Iran's intervention index in November 2020 had risen to first place among the six countries, because its mean response index has been the lowest since the COVID-19 outbreak and the index score has been ~50, the total number of confirmed cases is the highest in the six countries (Figures 3A–C). Therefore, early government and societal intervention is important to control the COVID-19 pandemic.

The total confirmed cases and daily growth rate of the COVID-19 pandemic were projected using machine learning under current intervention actions. Saudi Arabia, Pakistan, and Qatar will be flat in total confirmed cases and show a downward trend in the daily growth rate, while Iran will continue to rise in total confirmed cases and show no significant downward trend in the daily growth rate (Figures 5A–F). The daily confirmed case growth rates in Egypt, Pakistan, and Saudi Arabia are going to be <1 before March 2021. These projections show that although the total number of cases is still increasing, the number of daily confirmed cases or the daily confirmed case growth rate of COVID-19 in Saudi Arabia, Pakistan, Qatar, and Egypt shows a significant downward trend (Figures 2, 4, 5), indicating that these countries may significantly reduce daily confirmed cases and the COVID-19 pandemic will be controlled if current interventions are maintained or tightened; the cases in these countries still have the potential to rise if they are not properly controlled and intervened. However, the situation is still severe and needs to be strengthened further in Iran and Iraq.

This research has obtained some appropriate results, but the study has several limitations. First, this project or evaluation is based on the current intervention actions, but the current situation is not static because the government and societal intervention measures and control levels, such as whether to require masks, social distancing, and lockdowns, may change, and society also needs to resume work and gradual reopening (5, 6). It is also very important to note that the degree of societal compliance with these measures and regulations will determine the ultimate effectiveness of these interventions, but we did not analyze these because data on the extent of compliance with non-pharmaceutical measures are lacking and not comprehensive, which may have made the projection not very accurate. Therefore, the forecast results will also change as these interventions or societal compliance degree change. Even if the daily growth rate of some countries shows a downward trend, it is possible that the epidemic will re-erupt unless existing strict measures are strengthened and maintained at a high degree of compliance. Second, the total number of confirmed cases is not the actual number of infections daily and in total because it is impossible for all infected people to be reported or tested, especially in Eastern Mediterranean countries that do not trace contact and practice isolation of suspected and confirmed cases, so the confirmed cases in total and daily may be far below the true number (29–31). Finally, the projection and interpretation of the COVID-19 pandemic are challenging and should be carefully based on the situation of the COVID-19 pandemic and the above limitations (32). Unlike some studies that have predicted trends in COVID-19 (12, 14, 33), our study focused on changes and growth rate in the number of daily confirmed cases. However, it is believed that the evaluation and projection results are highly reliable because they are based not only on the number of cases but also on the shape of the pandemic curve to make predictions by machine learning (7, 9–11). These projections are useful in assessing the epidemic situation and taking appropriate intervention measures.

## Conclusion

The COVID-19 pandemic was evaluated and projected for six Eastern Mediterranean countries. The findings suggest that the number of active cases, daily confirmed cases, or daily confirmed-case growth rate of the COVID-19 pandemic in Egypt, Pakistan, Saudi Arabia, and Qatar showed a significant downward trend, which indicates that the COVID-19 pandemic will be basically under control in these countries, and the growth rate of daily confirmed cases may significantly reduce if current interventions are maintained or tightened, although these countries have the potential to rise if they are poorly controlled or intervened. Iran and Iraq may continue to rise in active cases and total confirmed cases with no significant downward trend in the daily growth rate, which indicates that one cannot be optimistic and the response must be further strengthened. It is hoped that these assessments and projections will contribute to a better response to the COVID-19 pandemic.

## Data Availability Statement

Publicly available datasets were analyzed in this study. This data can be found here: https://github.com/CSSEGISandData/COVID-19/tree/master/csse_covid_19_data.

## Author Contributions

YH proposed the idea and design of the study and had full access to all data in the study. WH, SA, and DH contributed to the raw data acquisition and writing of the manuscript. SA, WH, and YH contributed to important revisions of the manuscript. WH, SA, DH, SL, and YH contributed to the statistical analysis. All authors participated in data acquisition, data analysis, or data interpretation and reviewed and approved the final version.

## Conflict of Interest

The authors declare that the research was conducted in the absence of any commercial or financial relationships that could be construed as a potential conflict of interest.

## Supplementary Material

Supplementary Figure 1 Flowchart for methodology.

Supplementary Figure 2 Overall situation of the COVID-19 pandemic in Eastern Mediterranean countries: **(a–f)** overall situation of the COVID-19 pandemic in Iran, Saudi Arabia, Pakistan, Iraq, Qatar, and Egypt, respectively.

Supplementary Figure 3 Overall growth rates in total number of confirmed cases: **(a–f)** subgraph showing Iran, Saudi Arabia, Pakistan, Iraq, Qatar, and Egypt — exp model, a linear fit to an exponential law in log scale; glm-Poisson, a general linear regression Poisson model; GLM-gamma, a general linear regression gamma model method.

Supplementary Table 1 Machine-learning methods to forecast the trend of COVID-19.

Supplementary Material 1 R code for non-pharmaceutical intervention actions.

## Abbreviations

COVID-19: coronavirus disease 2019
WHO: The World Health Organization
OxCGRT: Oxford
COVID-19: Government Response Tracker
PI: 95% prediction interval
Yhat: each day in a future predicted value.
