# Supplementary figures and images for: Data-Driven and Machine-Learning Methods to Project Coronavirus Disease 2019 Pandemic Trend in Eastern Mediterranean
Source: Front Public Health. 2021 May 13;9:602353. doi: 10.3389/fpubh.2021.602353 (PMC8158576; doi:10.3389/fpubh.2021.602353)

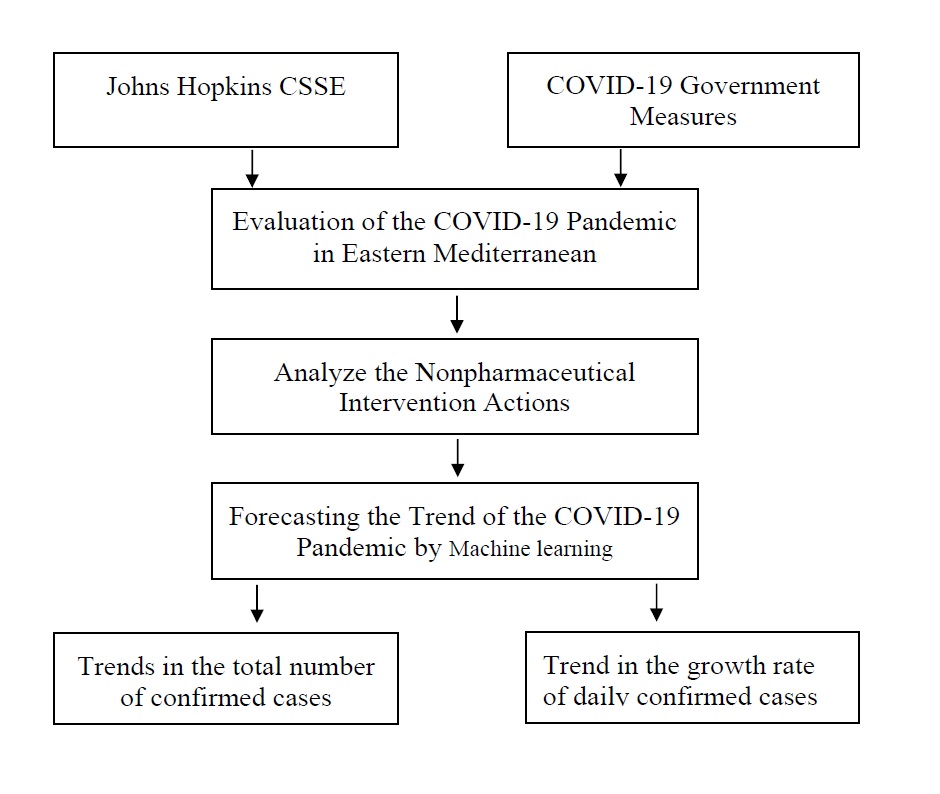

Supplement: Supplementary Figure 1 — Flowchart for methodology. [file Image_1.JPEG]

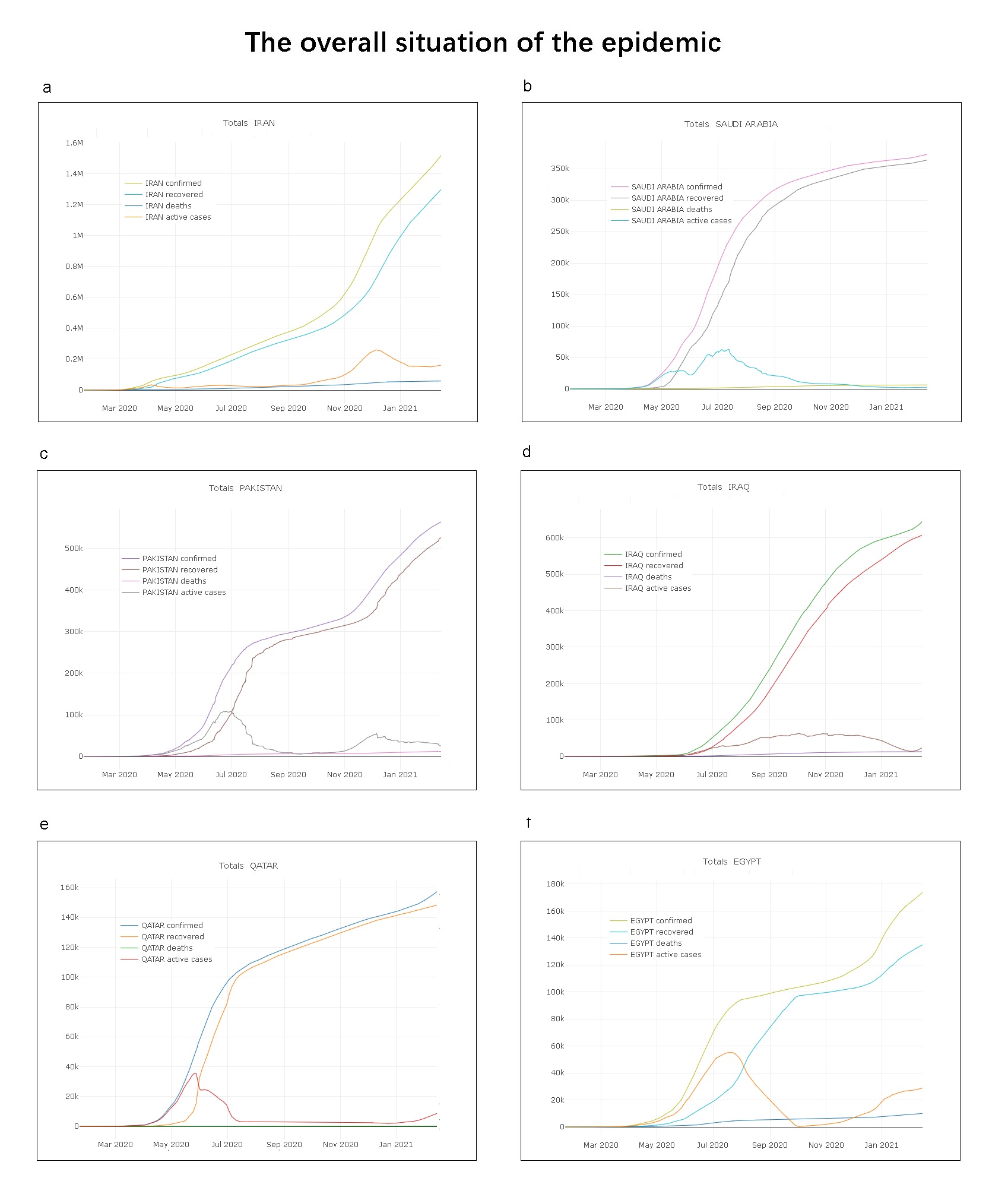

Supplement: Supplementary Figure 2 — Overall situation of the COVID-19 pandemic in Eastern Mediterranean countries: (a–f) overall situation of the COVID-19 pandemic in Iran, Saudi Arabia, Pakistan, Iraq, Qatar, and Egypt, respectively. [file Image_2.JPEG]

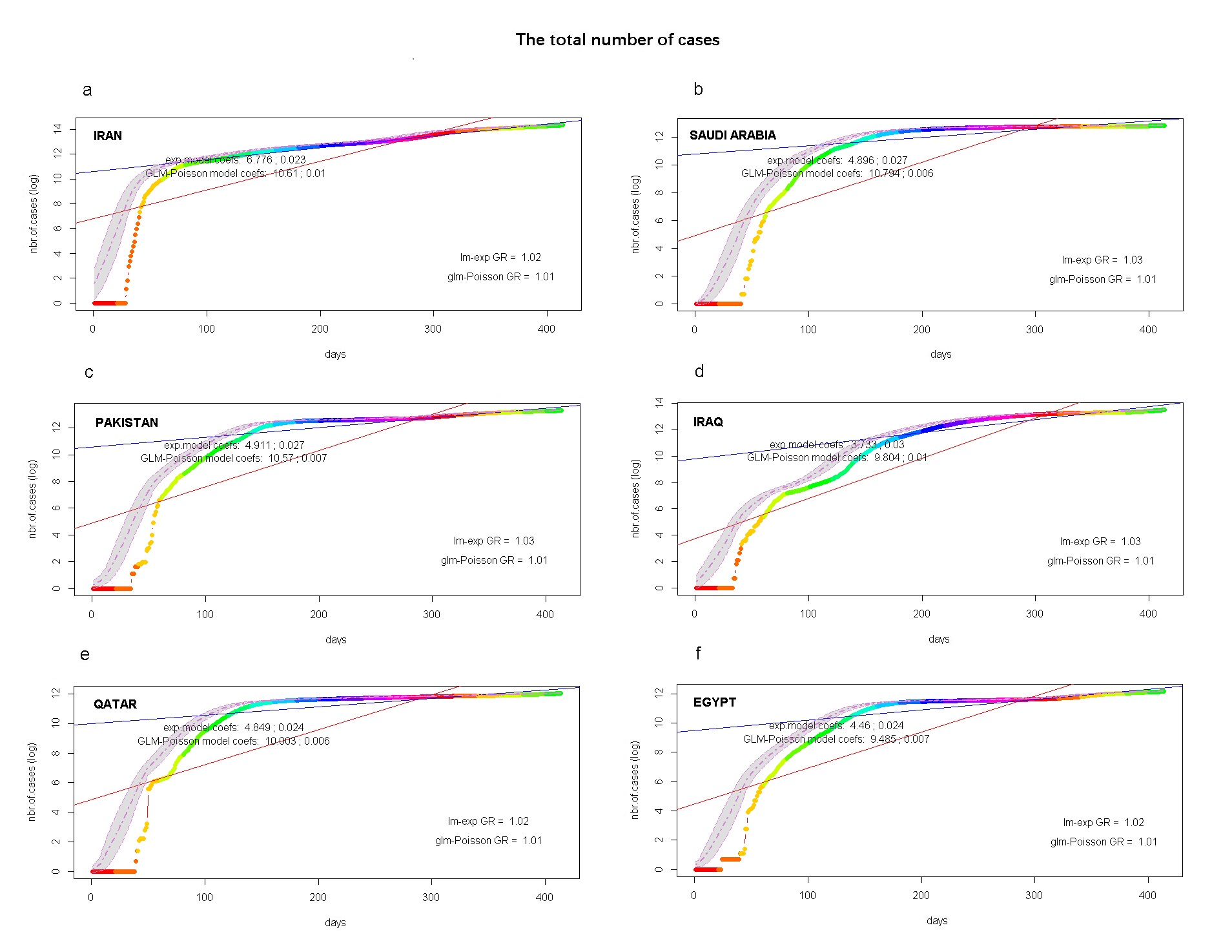

Supplement: Supplementary Figure 3 — Overall growth rates in total number of confirmed cases: (a–f) subgraph showing Iran, Saudi Arabia, Pakistan, Iraq, Qatar, and Egypt — exp model, a linear fit to an exponential law in log scale; glm-Poisson, a general linear regression Poisson model; GLM-gamma, a general linear regression gamma model method. [file Image_3.JPEG]
