# Supplementary material for: Data-Driven and Machine-Learning Methods to Project Coronavirus Disease 2019 Pandemic Trend in Eastern Mediterranean
Source: Front Public Health. 2021 May 13;9:602353. doi: 10.3389/fpubh.2021.602353 (PMC8158576; doi:10.3389/fpubh.2021.602353)
Supplement: Supplementary Material 1 — R code for non-pharmaceutical intervention actions. [file Data_Sheet_1.PDF]

```
#########
#########   1  Eastern Mediterranean ---- response index
#########

library(dslabs)
library(dplyr)
library(lubridate)
library(ggplot2)

# data download from https://ourworldindata.org/grapher/covid-stringency-index

data<-read.csv("covid-stringency-index.csv")
data$Entity
colnames(data)[4] <- 'Stringency_Index'

East_Medit <- c("Iran","Saudi Arabia","Pakistan","Iraq","Qatar","Egypt")
data1 =data[which(data $ Entity %in% East_Medit),]
rownames(data1) <- NULL
write.csv(data1, file="East_Medit.csv")

East_Medit1 <-read.csv("East_Medit.csv",header=T)

#######  1.1 Eastern Mediterranean --- boxplot and Violin Plot

## Violin Plot
p1 = ggplot(East_Medit1,aes(x =Entity, y = Stringency_Index,fill = Entity))
+geom_violin()
print(p1)

## Boxplot
p2 = ggplot(East_Medit1,aes(x = Entity, y = Stringency_Index,fill = Entity))
+geom_boxplot()+
  theme(axis.title.x  = element_text(face = 'italic'),
        axis.text.x =  element_text(angle = 45 , vjust = 0.5))
print(p2)

## Density Plot
p3 = ggplot(East_Medit1,aes(Stringency_Index,col=Entity)) +geom_density() +
  facet_wrap(~Entity,nrow = 2)  #nrow是显示行数
print(p3)

####### 1.2  Eastern Mediterranean 6 --- Nonpharmaceutical Intervention Actions

### "Iran","Saudi Arabia","Pakistan","Iraq","Qatar","Egypt"
```

```
Iran = filter(data,data$Entity=="Iran")
colnames(Iran)[4] <- 'Iran_Index'
Iran = Iran %>% select(Date,Iran_Index)

Saudi_Arabia = filter(data,data$Entity=="Saudi Arabia")
colnames(Saudi_Arabia)[4] <- 'Saudi_Arabia_index'
Saudi_Arabia = Saudi_Arabia %>% select(Date,Saudi_Arabia_index)

Pakistan = filter(data,data$Entity=="Pakistan")
colnames(Pakistan)[4] <- 'Pakistan_index'
Pakistan = Pakistan %>% select(Date,Pakistan_index)

Iraq = filter(data,data$Entity=="Iraq")
colnames(Iraq)[4] <- 'Iraq_Index'
Iraq = Iraq %>% select(Date,Iraq_Index)

Qatar = filter(data,data$Entity=="Qatar")
colnames(Qatar)[4] <- 'Qatar_Index'
Qatar = Qatar %>% select(Date,Qatar_Index)

Egypt = filter(data,data$Entity=="Egypt")
colnames(Egypt)[4] <- 'Egypt_Index'
Egypt = Egypt %>% select(Date,Egypt_Index)

# Define a function-multimerge
multimerge<-function(dat=list(),...){
  if(length(dat)<2)return(as.data.frame(dat))
  mergedat<-dat[[1]]
  dat[[1]]<-NULL
  for(i in dat){
    mergedat<-merge(mergedat,i,...,all=T)
  }
  return(mergedat)
}

East_Medit6<- multimerge(list(Iran,Saudi_Arabia, Pakistan, Iraq, Qatar, Egypt))


install.packages("lubridate")
library(lubridate)
East_Medit6$Date <- lubridate::dmy(East_Medit6$Date)

write.csv(East_Medit6, file="EM_Index.csv")
```

```
library(ggplot2)

vd = rbind(data.frame(v=East_Medit6$Date, y=East_Medit6$Iran_Index,
type=as.factor(1)),
          data.frame(v=East_Medit6$Date, y=East_Medit6$Saudi_Arabia_index,
type=as.factor(2)),
          data.frame(v=East_Medit6$Date, y=East_Medit6$Pakistan_index,
type=as.factor(3)),
          data.frame(v=East_Medit6$Date, y=East_Medit6$Iraq_Index,
type=as.factor(4)),
          data.frame(v=East_Medit6$Date, y=East_Medit6$Qatar_Index,
type=as.factor(5)),
          data.frame(v=East_Medit6$Date, y=East_Medit6$Egypt_Index,
type=as.factor(6)))

ggplot(vd,aes(x=v,y=y,shape=type,color=type,group=type))+geom_point()+labs(tit
le="Covid-19 Response Stringency Index")+xlab("Date")+ylab("Response
Stringency
Index")+scale_shape_manual(values=c(1,4,5,7,9,11))+scale_color_manual(values=c(1
:6))+theme(plot.title=element_text(hjust=0.5))+geom_line()
dev.off()

ggplot(vd,aes(x=v,y=y,shape=type,color=type,group=type))+labs(title="Covid-19
Response Stringency Index")+xlab("Date")+ylab("Response Stringency
Index")+scale_shape_manual(values=c(1,4,5,7,9,11))+scale_color_manual(values=c(1
:6))+theme(plot.title=element_text(hjust=0.5))+geom_line(size=1.0,shape=4)
dev.off()



#########
#########   2  Eastern Mediterranean --- Confirmed cases per million people
#########


# The data download from
https://github.com/owid/covid-19-data/tree/master/public/data/owid-covid-data.c
sv
data<-read.csv("owid-covid-data.csv")
data$location
colnames(data)[11] <- 'Cases_per_million'

East_Medit <- c("Iran","Saudi Arabia","Pakistan","Iraq","Qatar","Egypt")

data1 =data[which(data$location %in% East_Medit),]
```

```
rownames(data1) <- NULL
write.csv(data1, file="EM_permillon.csv")
EM_permillon1= data1

#######  2.1 Eastern Mediterranean - Violin Plot and Boxplot

## Violin Plot
p1 = ggplot(EM_permillon1,aes(x =location, y = Cases_per_million,fill = location))
+geom_violin()
print(p1)

## Boxplot
p2 = ggplot(EM_permillon1,aes(x = location, y = Cases_per_million,fill =location))
+geom_boxplot()+
  theme(axis.title.x  = element_text(face = 'italic'),
        axis.text.x =  element_text(angle = 45 , vjust = 0.5))
print(p2)

## Density Plot
p3 = ggplot(EM_permillon1,aes(Cases_per_million,col=location)) +geom_density() +

  facet_wrap(~location,nrow = 2)

#######  2.2  Eastern Mediterranean - The line chart

library(dplyr)

### "Iran","Saudi Arabia","Pakistan","Iraq","Qatar","Egypt"

data<-read.csv("owid-covid-data.csv")
colnames(data)[3] <- 'Entity'
colnames(data)[4] <- 'Date'

Iran = filter(data,data$Entity=="Iran")
colnames(Iran)[11] <- 'Iran_Index'
Iran = Iran %>% select(Date,Iran_Index)

Saudi_Arabia = filter(data,data$Entity=="Saudi Arabia")
colnames(Saudi_Arabia)[11] <- 'Saudi_Arabia_index'
Saudi_Arabia = Saudi_Arabia %>% select(Date,Saudi_Arabia_index)

Pakistan = filter(data,data$Entity=="Pakistan")
colnames(Pakistan)[11] <- 'Pakistan_index'
Pakistan = Pakistan %>% select(Date,Pakistan_index)
```

```
Iraq = filter(data,data$Entity=="Iraq")
colnames(Iraq)[11] <- 'Iraq_Index'
Iraq = Iraq %>% select(Date,Iraq_Index)

Qatar = filter(data,data$Entity=="Qatar")
colnames(Qatar)[11] <- 'Qatar_Index'
Qatar = Qatar %>% select(Date,Qatar_Index)

Egypt = filter(data,data$Entity=="Egypt")
colnames(Egypt)[11] <- 'Egypt_Index'
Egypt = Egypt %>% select(Date,Egypt_Index)


# multimerge
multimerge<-function(dat=list(),...){
  if(length(dat)<2)return(as.data.frame(dat))
  mergedat<-dat[[1]]
  dat[[1]]<-NULL
  for(i in dat){
    mergedat<-merge(mergedat,i,...,all=T)
  }
  return(mergedat)
}

EM_permillon6<- multimerge(list(Iran,Saudi_Arabia, Pakistan, Iraq, Qatar, Egypt))

write.csv(EM_permillon6, file="EM_permillon6.csv")

East_Medit6 EM_permillon6
#install.packages("lubridate")
library(lubridate)
East_Medit6$Date <- lubridate::dmy(East_Medit6$Date)

East_Medit6 <- East_Medit6[,-1]

library(ggplot2)
vd = rbind(data.frame(v=East_Medit6$Date, y=East_Medit6$Iran_Index,
type=as.factor(1)),
           data.frame(v=East_Medit6$Date, y=East_Medit6$Saudi_Arabia_index,
type=as.factor(2)),
           data.frame(v=East_Medit6$Date, y=East_Medit6$Pakistan_index,
type=as.factor(3)),
           data.frame(v=East_Medit6$Date, y=East_Medit6$Iraq_Index,
type=as.factor(4)),
           data.frame(v=East_Medit6$Date, y=East_Medit6$Qatar_Index,
```

```
type=as.factor(5)),
          data.frame(v=East_Medit6$Date, y=East_Medit6$Egypt_Index,
type=as.factor(6)))

ggplot(vd,aes(x=v,y=y,shape=type,color=type,group=type))+geom_point()+labs(tit
le="Total confirmed cases of COVID-19 per million people
")+xlab("Date")+ylab("Total confirmed cases per
million")+scale_shape_manual(values=c(1,4,5,7,9,11))+scale_color_manual(values=c(
1:6))+theme(plot.title=element_text(hjust=0.5))+geom_line()

ggplot(vd,aes(x=v,y=y,shape=type,color=type,group=type))+labs(title="Total
confirmed cases of COVID-19 per million people")+xlab("Date")+ylab("Total
confirmed cases per
million")+scale_shape_manual(values=c(1,4,5,7,9,11))+scale_color_manual(values=c(
1:6))+theme(plot.title=element_text(hjust=0.5))+geom_line(size=1.5,shape=4)
dev.off()



#########
#########   3  Eastern Mediterranean --- response index and the number of
confirmed   #########

library(dslabs)
library(dplyr)
library(lubridate)
library(ggplot2)

data<-read.csv("owid-covid-data.csv")

East_Medit <- c("Iran","Saudi Arabia","Pakistan","Iraq","Qatar","Egypt")

data1 =data[which(data$location %in% East_Medit),]
rownames(data1) <- NULL
data1 = data1 %>% select(location,date,total_cases)
colnames(data1)[1] <- 'Entity'
colnames(data1)[2] <- 'Date'
write.csv(data1, file="EM_totalcase.csv")
EM_totalcase1 <- data1

#######  3.1 Eastern Mediterranean --- Violin Plot and Boxplot for Total number
of confirmed cases

## Violin Plot
p1 = ggplot(EM_totalcase1,aes(x =Entity, y = total_cases,fill = Entity))
```

```
+geom_violin()
print(p1)

## Boxplot
p2 = ggplot(EM_totalcase1,aes(x = Entity, y = total_cases,fill = Entity))
+geom_boxplot()+
  theme(axis.title.x  = element_text(face = 'italic'),
        axis.text.x =  element_text(angle = 45 , vjust = 0.5))
print(p2)

## Density Plot
p3 = ggplot(EM_totalcase1,aes(total_cases,col=Entity)) +geom_density() +
  facet_wrap(~Entity,nrow = 2)
print(p3)

####### 1.2  Eastern Mediterranean --- Total number of confirmed cases

library(dplyr)
data <- EM_totalcase1
### "Iran","Saudi Arabia","Pakistan","Iraq","Qatar","Egypt"

Iran = filter(data,data$Entity=="Iran")
colnames(Iran)[4] <- 'Iran_total'
Iran = Iran %>% select(Date,Iran_total)

Saudi_Arabia = filter(data,data$Entity=="Saudi Arabia")
colnames(Saudi_Arabia)[4] <- 'Saudi_Arabia_total'
Saudi_Arabia = Saudi_Arabia %>% select(Date,Saudi_Arabia_total)

Pakistan = filter(data,data$Entity=="Pakistan")
colnames(Pakistan)[4] <- 'Pakistan_total'
Pakistan = Pakistan %>% select(Date,Pakistan_total)

Iraq = filter(data,data$Entity=="Iraq")
colnames(Iraq)[4] <- 'Iraq_total'
Iraq = Iraq %>% select(Date,Iraq_total)

Qatar = filter(data,data$Entity=="Qatar")
colnames(Qatar)[4] <- 'Qatar_total'
Qatar = Qatar %>% select(Date,Qatar_total)

Egypt = filter(data,data$Entity=="Egypt")
colnames(Egypt)[4] <- 'Egypt_total'
Egypt = Egypt %>% select(Date,Egypt_total)
```

```
# multimerge
multimerge<-function(dat=list(),...){
  if(length(dat)<2)return(as.data.frame(dat))
  mergedat<-dat[[1]]
  dat[[1]]<-NULL
  for(i in dat){
    mergedat<-merge(mergedat,i,...,all=T)
  }
  return(mergedat)
}

EM_totalcase6<- multimerge(list(Iran,Saudi_Arabia, Pakistan, Iraq, Qatar, Egypt))

write.csv(EM_totalcase6, file="EM_totalcase6.csv")


####### 3.3  relationship between government response index and the number of confirmed cases


EM_Index <- read.csv("EM_Index.csv")
EM_Index<- EM_Index[,-1]
EM_Index_mean <- apply(EM_Index[1:419,2:7], 2, mean,na.rm=T)
EM_Index_mean

#Iran_Index Saudi_Arabia_index    Pakistan_index       Iraq_Index       Qatar_Index
    Egypt_Index
#51.80103          55.24105          54.81881          62.92697          60.56647
54.73883

EM_Index_Median <- apply(EM_Index[1:419,2:7], 2, median,na.rm=T)
EM_Index_Median

#Iran_Index Saudi_Arabia_index    Pakistan_index       Iraq_Index       Qatar_Index
    Egypt_Index
#58.80            57.41            58.80            61.11            64.81            62.96

class(EM_Index_Median)

Index_mean = matrix(EM_Index_mean)
Index_Median = matrix(EM_Index_Median)

Entity = c("Iran","Saudi Arabia","Pakistan","Iraq","Qatar","Egypt")

EM_Total = EM_totalcase6
```

```
Total_cases = EM_Total[392,]
Total_cases = Total_cases[,-1]
class(Total_cases)
Total_cases
colnames(Total_cases) <- NULL
rownames(Total_cases) <- NULL
#unlist(Total_cases)
Total_cases
Total_cases <- t(Total_cases)

Casepermillion = EM_permillon6
Casepermillion = Casepermillion[392,]
Casepermillion = Casepermillion[,-1]
class(Casepermillion)
Casepermillion
colnames(Casepermillion) <- NULL
rownames(Casepermillion) <- NULL
#unlist(Total_cases)
Casepermillion
Casepermillion<- t(Casepermillion)
Casepermillion

lastday_index = EM_Index[418,]
lastday_index = lastday_index[,-1]
class(lastday_index)
lastday_index
colnames(lastday_index) <- NULL
rownames(lastday_index) <- NULL
#unlist(Total_cases)
lastday_index
lastday_index<- t(lastday_index)
lastday_index

Entity <- matrix(Entity)

mix=
data.frame(Entity,Total_cases,Casepermillion,lastday_index,Index_mean,Index_Media
n)

class(mix)

write.csv(mix, file=" EM_Index and Case.csv")

#index and total number of confirmed cases
ggplot(mix,aes(x=mix$Total_cases,y=mix$lastday_index))+geom_point(stat="identit
```

```
y",fill="steelblue",colour="steelblue",width = 1.8,size=8.5)+labs(title="Relationship between Total confirmed cases of COVID-19 and Response Index")+xlab("Total confirmed cases of COVID-19")+ylab("Response Index")+theme(plot.title=element_text(hjust=0.5))+geom_text(aes(label = mix$Entity, vjust = -1.2, hjust = 0.0), show.legend = TRUE)

# Index_mean and total number of confirmed cases
ggplot(mix,aes(x=mix$Total_cases,y=mix$Index_mean))+geom_point(stat="identity",fill="green",colour="green",width = 1.8,size=8.5)+labs(title="Relationship between Total confirmed cases of COVID-19 and Response Index")+xlab("Total confirmed cases of COVID-19")+ylab("Response Index")+theme(plot.title=element_text(hjust=0.5))+geom_text(aes(label = mix$Entity, vjust = -0.9, hjust =  0.0), show.legend = TRUE)

# Index_median and total number of confirmed cases
ggplot(mix,aes(x=mix$Total_cases,y=mix$Index_Median))+geom_point(stat="identity",fill="purple",colour="purple",width = 1.8,size=8.5)+labs(title="Relationship between Total confirmed cases of COVID-19 and Response Index")+xlab("Total confirmed cases of COVID-19")+ylab("Response Index")+theme(plot.title=element_text(hjust=0.5))+geom_text(aes(label = mix$Entity, vjust = 1.8, hjust = 0.0), show.legend = TRUE)

# Index and total number of confirmed cases per million
ggplot(mix,aes(x=mix$Casepermillion,y=mix$lastday_index))+geom_point(stat="identity",fill="steelblue",colour="steelblue",width = 1.8,size=8.5)+labs(title="Relationship between the cases per million and Response Index")+xlab("Cases per million")+ylab("Response Index")+theme(plot.title=element_text(hjust=0.5))+geom_text(aes(label = mix$Entity, vjust = -1.2, hjust = 0.0), show.legend = TRUE)

# Index_mean and total number of confirmed cases per million
ggplot(mix,aes(x=mix$Casepermillion,y=mix$Index_mean))+geom_point(stat="identity",fill="green",colour="green",width = 1.8,size=8.5)+labs(title="Relationship between the cases per million and Response Index")+xlab("Cases per million")+ylab("Response Index")+theme(plot.title=element_text(hjust=0.5))+geom_text(aes(label = mix$Entity, vjust = -1.2, hjust =  0.0), show.legend = TRUE)

# Index_median and total number of confirmed cases per million
ggplot(mix,aes(x=mix$Casepermillion,y=mix$Index_Median))+geom_point(stat="identity",fill="purple",colour="purple",width = 1.8,size=8.5)+labs(title="Relationship between the cases per million and Response Index")+xlab("Cases per million")+ylab("Response Index")+theme(plot.title=element_text(hjust=0.5))+geom_text(aes(label = mix$Entity, vjust = -1.2, hjust =  0.0), show.legend = TRUE)
```
